# Supplementary material for: A deep learning framework identifies dimensional representations of Alzheimer’s Disease from brain structure
Source: Nat Commun. 2021 Dec 3;12:7065. doi: 10.1038/s41467-021-26703-z (PMC8642554; doi:10.1038/s41467-021-26703-z)
Supplement: Supplementary file 1 — Supplementary Information [file 41467_2021_26703_MOESM1_ESM.pdf]

Supplement to:

A deep learning framework identifies dimensional representations of Alzheimer's Disease from brain structure

Authors: Zhijian Yang, Ilya M. Nasrallah, Haochang Shou, Junhao Wen, Jimit Doshi, Mohamad Habes, Guray Erus, Ahmed Abdulkadir, Susan M. Resnick, Marilyn S. Albert, Paul Maruff, Jurgen Fripp, John C. Morris, David A. Wolk, Christos Davatzikos

1. SUPPLEMENTARY METHODS

1.1 Smile-GAN Regularization.

Regularizations mentioned in this section serve to constrain the function class where the mapping function f is sampled from, so that it is truly meaningful while matching the distribution. The change loss is to control the distance of transformations. We assume that only some specific regions will be affected as disease progresses along each direction, which means that the true underlying transformation only changes some regions while keeping the rest unchanged. To encourage sparsity, we define the change loss to be the l_1 distance between the synthesized patient (PT) data and the original cognitively normal (CN) participant data:

$$L_{change}(\mathrm{f}) = E_{\mathbf{x}\sim p_{CN},\mathbf{z}\sim p_{Sub}}[\|\mathrm{f}(\mathbf{x},\mathbf{z}) - \mathbf{x}\|_1] \tag{1}$$

The rest regularizations are based on Lipschitz continuity of the mapping function f and clustering function g. First, with function f being K-Lipschitz continuous, we have that, for fixed Sub variable $\mathbf{z} = \mathbf{a}$ and $\forall \mathbf{x}_1, \mathbf{x}_2 \in X$, $\|\mathrm{f}(\mathbf{x}_1, \mathbf{a}) - \mathrm{f}(\mathbf{x}_2, \mathbf{a})\|_2 \leq K_1\|\mathbf{x}_1 - \mathbf{x}_2\|_2$. Thus, by controlling the constant K_1, the same mapping direction will preserve original distances among CN data by transforming them into a compact cluster but not scattering the synthesized PT data. Moreover, with function g being K-Lipschitz continuous, we derive that, $\forall \mathbf{z}_1, \mathbf{z}_2 \sim p_{Sub}$, $\mathbf{z}_1 \neq \mathbf{z}_2$ and $\bar{\mathbf{x}} \sim p_{CN}$, $\|\mathrm{f}(\bar{\mathbf{x}}, \mathbf{z}_1) - \mathrm{f}(\bar{\mathbf{x}}, \mathbf{z}_2)\|_2$ is lower-bounded by $\frac{\sqrt{2}}{K_2} - \frac{1}{K_2}(\|\mathrm{g}(\mathrm{f}(\bar{\mathbf{x}}, \mathbf{z}_1)) - \mathbf{z}_1\|_2 + \|\mathrm{g}(\mathrm{f}(\bar{\mathbf{x}}, \mathbf{z}_2)) - \mathbf{z}_2\|_2)$:

$$\|\mathrm{f}(\bar{\mathbf{x}}, \mathbf{z}_1) - \mathrm{f}(\bar{\mathbf{x}}, \mathbf{z}_2)\|_2 \geq \frac{1}{K_2}\left(\|\mathrm{g}(\mathrm{f}(\mathbf{x}, \mathbf{z}_1)) - \mathrm{g}(\mathrm{f}(\bar{\mathbf{x}}, \mathbf{z}_2))\|_2\right) \tag{2}$$

$$\geq \frac{1}{K_2}\left(\|\mathbf{z}_1 - \mathbf{z}_2\|_2 - \|\mathrm{g}(\mathrm{f}(\bar{\mathbf{x}}, \mathbf{z}_1)) - \mathbf{z}_1\|_2 - \|\mathrm{g}(\mathrm{f}(\bar{\mathbf{x}}, \mathbf{z}_2)) - \mathbf{z}_2\|_2\right) \tag{3}$$

$$= \frac{\sqrt{2}}{K_2} - \frac{1}{K_2}\left(\|\mathrm{g}(\mathrm{f}(\bar{\mathbf{x}}, \mathbf{z}_1)) - \mathbf{z}_1\|_2 + \|\mathrm{g}(\mathrm{f}(\bar{\mathbf{x}}, \mathbf{z}_2)) - \mathbf{z}_2\|_2\right) \tag{4}$$

Therefore, we can control differences among mapping directions to be non-trivial (i.e., same CN data is mapped to significantly different PT data along distinct directions) by minimizing the distance between sampled Sub variable $\mathbf{z}$ and reconstructed Sub variable $\mathrm{g}(\mathrm{f}(\mathbf{x},\mathbf{z}))$. We, thus, define another cluster loss to be the cross-entropy between sampled z and reconstructed $\mathrm{g}(\mathrm{f}(\mathbf{x},\mathbf{z}))$. By denoting $l(\mathbf{a},\mathbf{b}) = -\sum_{i=1}^{k} \mathbf{a}^i \log \mathbf{b}^i$, we can write the cluster loss as:

$$L_{cluster}(\mathrm{f},\mathrm{g}) = E_{\mathbf{x}\sim p_{CN},\mathbf{z}\sim p_{Sub}}\left[l\left(\mathbf{z},\mathrm{g}\big(\mathrm{f}(\mathbf{x},\mathbf{z})\big)\right)\right] \quad (5)$$

Function g here is considered as the approximation of posterior distribution $P(\mathbf{z}|\mathrm{f}(\mathbf{x},\mathbf{z}))$. Therefore, minimization of cross entropy-loss can be also interpreted as maximizing the mutual information between synthesized PT variable $\mathbf{y}'$ and Sub variable $\mathbf{z}$ as shown in Remark 1. In this sense, the mapping function is forced to best utilize information of Sub variable while also keeping mutual information between transformed data and original CN data by controlling transformation distance.

Moreover, with all constraints imposed on function f and the assumption $p_{PT} = p_{\mathrm{f}}$, we consider that f satisfies all necessary conditions and is a good approximation of the underlying function h such that $\mathrm{f}(\mathbf{x},\mathbf{z}) \approx \mathrm{h}\big(\mathbf{x},\sigma(\mathbf{z})\big)$ for some $\sigma \in \Omega$, where Ω is the class of all permutation functions which changes the order of M elements in vector $\mathbf{z}$. By reordering the results given by models, we simply ignore the permutation function and write as $\mathrm{f}(\mathbf{x},\mathbf{z}) \approx \mathrm{h}(\mathbf{x},\mathbf{z})$, without loss of generality. For any new PT data $\mathbf{y}_i = \mathrm{h}(\mathbf{x}_i,\mathbf{z}_i) \sim p_{PT}$ coming in, we can have $\mathrm{g}(\mathbf{z}_i) = \mathrm{g}(\mathrm{h}(\mathbf{x}_i,\mathbf{z}_i)) \approx \mathrm{g}(\mathrm{f}(\mathbf{x}_i,\mathbf{z}_i))$, whose results were trained to be close to $\mathbf{z}_i$. Therefore, function g can be used as a clustering function on unseen PT data.

Remark 1. By minimizing the cluster loss $L_{cluster}$ defined above, we are maximizing a lower bound of the mutual information between Sub variable z and synthesized PT data $\mathbf{y}' = \mathrm{f}(\mathbf{x},\mathbf{z})$. Considering $Q(\mathbf{z}|\mathbf{y}') = \mathrm{g}(\mathbf{y}')$ to be an approximation of distribution $P(\mathbf{z}|\mathrm{f}(\mathbf{x},\mathbf{z}))$, mutual information denoted by I and entropy denoted by H, we can derive that:

$$I\big(\mathbf{z};\mathrm{f}(\mathbf{x},\mathbf{z})\big) = H(\mathbf{z}) - H\big(\mathbf{z}\big|\mathrm{f}(\mathbf{x},\mathbf{z})\big) \quad (6)$$

$$= E_{\mathbf{y}'\sim \mathrm{f}(\mathbf{x},\mathbf{z})}\left[E_{\mathbf{z}'\sim P(\mathbf{z}|\mathbf{y}')}[\log P(\mathbf{z}'|\mathbf{y}')]\right] + H(\mathbf{z}) \quad (7)$$

$$= E_{\mathbf{y}'\sim \mathrm{f}(\mathbf{x},\mathbf{z})}\left[D_{KL}(P(\cdot\,|\mathbf{y}')||Q(\cdot\,|\mathbf{y}')) + E_{\mathbf{z}'\sim P(\mathbf{z}|\mathbf{y}')}[\log Q(\mathbf{z}'|\mathbf{y}')]\right] + H(\mathbf{z}) \quad (8)$$

$$\geq E_{\mathbf{y}'\sim \mathrm{f}(\mathbf{x},\mathbf{z})}\left[E_{\mathbf{z}'\sim P(\mathbf{z}|\mathbf{y}')}[\log Q(\mathbf{z}'|\mathbf{y}')]\right] \quad (9)$$

$$= E_{\mathbf{z}\sim p_{Sub},\mathbf{y}'\sim \mathrm{f}(\mathbf{x},\mathbf{z})}[\log Q(\mathbf{z}|\mathbf{y}')] \quad (10)$$

$$= E_{\mathbf{z}\sim p_{Sub},\mathbf{x}\sim p_{CN}}\left[\log Q\big(\mathbf{z}\big|\mathrm{f}(\mathbf{x},\mathbf{z})\big)\right] \quad (11)$$

$$\approx \frac{1}{nm}\sum_{i=1}^{n}\sum_{j=1}^{m}\langle \mathbf{z}_i, \log \mathrm{g}\left(\mathrm{f}\big(\mathbf{x}_j,\mathbf{z}_i\big)\right)\rangle \quad (12)$$

The fifth and sixth line follows the Lemma 5.1 in Info-GAN[1] and law of the unconscious statistician (Lotus) Theorem respectively. Therefore, we have the mutual information bounded

below by $\frac{1}{nm}\sum_{i=1}^{n}\sum_{j=1}^{m}\langle \mathbf{z}_i, \log \mathrm{g}\left(\mathrm{f}(\mathbf{x}_j, \mathbf{z}_i)\right)\rangle$. Maximization of this lower bound is equivalent to minimizing the cluster loss $L_{cluster}$.

1.2 Smile-GAN Implementation Details

1.2.1 Network Architecture

To improve the rate of convergence, the mapping function, instead of directly transforming the CN data to the synthesized PT data, first learns a change in the CN data and then takes the sum of them to obtain the synthesized PT data. Therefore, the architecture of the mapping function f can be divided into two phases as shown in main Fig. 7C. In the first phase, the CN data and the Sub variable are mapped to latent representations with the same dimension through encoder and decoder, respectively. The second phase has one decoding structure mapping the dot-product of two representations to the change $\tilde{\mathbf{x}}$, which is added to the CN data $\mathbf{x}$ to generate the synthesized PT data. The discriminator D and the clustering function g have similar encoding structures, with D mapping PT/synthesized PT data to prediction vector with dimension 2 while the encoder g mapping the synthesized PT data to a Sub representation. More details are shown in Supplementary Table1 and Supplementary Table2.

Supplementary Table1: Architecture of mapping function f

	Layer	Input Size	Bias Term	Leaky Relu α	Output Size
Phase1 (Encoder)	Linear1+Leaky-Relu	145*1	No	0.2	72*1
	Linear2+Leaky-Relu	72*1	No	0.2	36*1
Phase1 (Decoder)	Linear1+Sigmoid	M*1	Yes	NA	36*1
Phase2	Linear1+Leaky-Relu	36*1	No	0.2	72*1
	Linear2+Leaky-Relu	72*1	No	0.2	145*1
	Linear3	145*1	No	NA	145*1

Supplementary Table 2: Architecture of discriminator D and clustering function g

	Layer	Input Size	Bias Term	Leaky Relu α	Output Size
Discriminator	Linear1+Leaky-Relu	145*1	Yes	0.2	72*1
	Linear2+Leaky-Relu	72*1	Yes	0.2	36*1
	Linear3+Softmax	36*1	Yes	NA	2*1
Clustering	Linear1+Leaky-Relu	145*1	Yes	0.2	145*1
	Linear2+Leaky-Relu	145*1	Yes	0.2	72*1
	Linear3+Leaky-Relu	72*1	Yes	0.2	36*1
	Linear4+Softmax	36*1	Yes	NA	M*1

1.2.2 Training Details

We ensure Lipschitz continuity of functions f and g by performing weight clipping[2]. With Θ_{g} and Θ_{f} representing the space where weights of function θ_{f} and θ_{g} lie in, the compactness of Θ_{g} and Θ_{f} implies the K-Lipschitz continuity of functions f and g, where Lipschitz constants K_1 and K_2 only depend on Θ_{g} and Θ_{f}. The compactness is achieved by clapping the weight space to a fixed box ($\Theta = [-c, c]^d$). In the implementation, c is empirically chosen to be 0.5 for both Θ_{g} and Θ_{f}.

However, different bounds for f and g or relaxation of bounds does not make much difference to results.

We set two parameters to be $\mu = 5$ and $\lambda = 9$ for all experiments. Also, we performed gradient clip for each iteration to avoid the explosion of gradient during the training process. For optimization, we used ADAM optimizer[3] with learning rate 0.0004 for Discriminator D and 0.002 for mapping f and clustering function g. β_1 and β_2 are 0.5 and 0.999, respectively.

1.2.3 Algorithm

Detailed training procedure of Smile-GAN is disclosed by Algorithm 1.

Algorithm 1: Smile-GAN training procedure. l_c represents cross entropy loss and e_i represents a one hot vector with 1 at i_{th} component.

while *not meeting stopping criteria or reaching max_epoch* **do**
  for *all batches* $\{\mathbf{x}_i\}_{i=1}^{m}, \{\mathbf{y}_i\}_{i=1}^{m}$ **do**
    Sample m integers $\{a_i\}_{i=1}^{m}$ *with* $a_i \sim$ *discrete-U* $(1, M)$ *and let* $\mathbf{z}_i = \mathbf{e}_{a_i}$
    Update weights of discriminator D: Use ADAM to update θ_D with gradient:
    $\nabla_{\theta_\mathrm{D}} \frac{1}{m}\sum_{i=1}^{m}[(l_c(\mathrm{D}(y_i), \mathbf{e}_1) + l_c(\mathrm{D}(\mathrm{f}(\mathbf{x}_i, \mathbf{z}_i), \mathbf{e}_0)))]$
    Update weights of mapping function f: Use ADAM to update θ_f with gradient:
    $\nabla_{\theta_\mathrm{f}} \frac{1}{m}\sum_{i=1}^{m}[(l_c(\mathrm{D}(\mathrm{f}(\mathbf{x}_i, \mathbf{z}_i), \mathbf{e}_1) + \lambda l_c(\mathrm{g}(\mathrm{f}(\mathbf{x}_i, \mathbf{z}_i), \mathbf{z}_i) + \mu\|\mathrm{f}(\mathbf{x}_i, \mathbf{z}_i) - \mathbf{x}_i\|_1)]$
    Update weights of clustering function g: Use ADAM to update θ_g with gradient:
    $\nabla_{\theta_\mathrm{g}} \frac{1}{m}\sum_{i=1}^{m}[(\mathrm{g}(\mathrm{f}(\mathbf{x}_i, \mathbf{z}_i), \mathbf{z}_i)]$
    $(\theta_\mathrm{f}, \theta_\mathrm{g}) = clip((\theta_\mathrm{f}, \theta_\mathrm{g}), -c, c)$
  end
end

1.2.4 Stopping Criteria

For the real application, since the ground truth of patterns is unknown, we adopt an approximation of the Wasserstein distance (WD) as one metric for monitoring the training process and choosing the stopping point. For Smile-GAN, instead of deriving WD from optimization, we used the closed-form formula to compute the distance. For stopping criteria, we assume that, in the CN group and in all subpopulations of the PT group, the lower-dimensional representation of each data point (ROIs) is sampled from a multivariate Gaussian distribution. Though this assumption might be strong, it does enable us to estimate the WD quickly.

To be more specific, for each of M Sub variables, $\mathbf{z} = \mathbf{z}_i$ and for all samples in CN group $\mathbf{X} = \{\mathbf{x}_1, \mathbf{x}_2, \cdots, \mathbf{x}_n\}$, we calculate the mean vector m_1^i and covariance matrix $\mathbf{C}_1^i$ of $\mathrm{f}(\mathbf{X}, \mathbf{z}_i)$. Also, from samples in PT group Y, we take out the subset $Y_i = \{\mathbf{y}_j\}^i$ such that $\mathrm{g}(\mathbf{y}_j)$ has highest value at position i for all $\mathbf{y}_j \in Y^i$. For this subset, we calculate the mean vector $\mathbf{m}_2^i$ and covariance matrix

$\mathbf{C}_2^i$. With mean vectors and covariance matrices, we can compute the 2nd Wasserstein distance using the formula for two multivariate gaussian measure:

$$W_2\left(\mu_{\mathrm{f}(\mathbf{X},\mathbf{z}_i)}, \mu_{Y_i}\right) = \left\|\mathbf{m}_1^i - \mathbf{m}_2^i\right\|_2^2 + \mathrm{trace}(\mathbf{C}_1^i + \mathbf{C}_2^i - 2\left(\mathbf{C}_2^{i\frac{1}{2}}\mathbf{C}_1^i\mathbf{C}_2^{i\frac{1}{2}}\right)^{\frac{1}{2}}) \tag{13}$$

If we further assume that all features are independent, we can derive diagonal covariance matrices which make the computation even faster. Based on our experiments on synthetic and semi-synthetic datasets, these assumptions do not affect monitoring the training process.

Moreover, to deal with cases when inconsistencies exist between WD and model performance, we also derive two other metrics: alteration quantity (AQ), which represents the number of participants whose dominated pattern type alter in the last five epochs. A small AQ represents high stability of the model. Lastly, the cluster loss, indicating the performance of clustering function g, is also considered as part of the stopping criteria.

1.3 Validation of Smile-GAN model

1.3.1 Synthetic Test

Simulated data were generated with equal dimension to the number of ROIs (145 ROIs). For each participant, the 145 ROIs were simulated by sampling from a normal distribution N (1, 0.1). In total, 1200 participants were generated independently and then randomly split into two half-split sets, with each (600) being CN and pseudo-PT group, respectively. The atrophy simulation was only introduced for pseudo-PT participants, which were further divided into 3 pattern types with the same number of participants (200). For each pattern type, the values of specific pre-selected ROIs were decreased by 20%. Moreover, to simulate confounding non-disease-related effects, we randomly sampled 200 participants from both CN and pseudo-PT respectively and decreased the values by 40% in some other ROIs that were not among those pre-selected for the disease pattern which make the confounding factors severe – much stronger than the simulated disease-related patterns. The simulation ground truth for the confounding patterns and the 3 pattern types are shown in Supplementary fig. 1(A) (i) and (ii), respectively. Note that overlapping of ROIs across patterns was imposed to better follow the nature of atrophy.

To add variability of the clustering performance, we repeated the simulated data generation and ran the experiment independently 20 times. We first checked the clustering accuracy and validated the potential of WD for monitoring the raining process. Then we investigated the ROIs captured by mapping functions f along different directions. We calculated the mean difference between CN and synthesized PT generated along K mapping directions and inspected ROIs with a significant decreasing in values.

1.3.2 Semi-synthetic Test

We selected 526 CN from ADNI 1 and 2 databases and included their 145 ROI volumes derived from baseline T1 MRI. CN participants are split into two sets, 200 as the CN group, 326 as the pseudo-PT group. 326 pseudo-PT participants are further equally divided into three sets and different levels of atrophy were introduced to selected GM regions by artificially decreasing ROI volumes. Atrophy in medial temporal lobe was introduced to the first set. Atrophy in selected cortical regions was introduced to the second set. For the third set, a combination of these two patterns was introduced. The effects of differences in severity of atrophy were tested by decreasing the chosen ROIs' volume by 30%, 10-30% or 10-20% (uniformly sampled within the range) in separate experiments. By starting from actual CN participant data, semi-synthetic data should incorporate more realistic non-disease-related variations among pseudo-PT groups for validation.

We first validated the potential of WD for monitoring the training process on semi-synthetic data whose distribution is closer to the distribution of real dataset which may violate the assumption we made for stopping criteria. Second, we compared the performance of Smile-GAN with two other semi-supervised methods, HYDRA[4] and CHIMERA[5], and also with two basic clustering methods, K-means and Gaussian mixture modeling (GMM) on these same tasks using similar hold-out validation. Each time running the model, we randomly split 80% data as the training set. For one hold-out analysis, we repeat this procedure C times and the final clustering membership was determined by a consensus clustering strategy across models trained on C different splits. Because of differences in required training time, C is chosen to be 5 for the Smile-GAN model, but 50 for other methods to maximize their performance. For each model, we performed hold-out analysis 10 times and reported the mean and standard deviation of clustering accuracies. Training data were normalized as required for each model. For two clustering methods, K-means and GMM, PT data were first normalized with respect to CN data and then utilized to train the model. Since K-means and GMM do not have equal access to CN data as other three semi-supervised methods, we implemented one additional variant of these two methods according to Dong et al.[5]. We computed a "distance" for each PT participant: we computed the difference vector between each PT point and its Euclidean nearest neighbor in the CN group. These distances were clustered instead of the original PT data. For implementation of HYDRA, we used the online PyPI package pyHYRDA 1.0.8. For Chimera, we used the GitHub package (https://github.com/aoyandong/CHIMERA), which may be lack of maintenance. K-means and GMM were both implemented via the package scikit-learn 0.24.2 with 400 initial points and 500 max-iterations; convergence thresholds for both K-means and GMM were set to be 1e-5. For GMM, K-means was chosen as the method for weight initialization and covariance type was set to be 'full'.

1.3.3 Rare Pattern and Non-uniform Z Experiment

The same procedure introduced in Supplementary section 1.3.2 was used to construct new CN and pseudo-PT groups, except that instead of equally dividing the pseudo-PT group, we introduced the second type of atrophy (i.e. atrophy in cortical regions) to a smaller fraction of participants while keeping sizes of other two groups the same. With the size of the pseudo-PT group fixed, we simulated the rare pattern at 33% (standard), 20%, 15%, and 10% of the pseudo-PT group respectively and tested the performance of Smile-GAN through the same hold-out validation introduced above. Moreover, to test robustness of Smile-GAN model to non-uniform distribution

of variable Z, we set the distribution of variable Z to be P(Z=1)=1/6, P(Z=2)=1/3 and P(Z=3)=1/2 and repeated the Semi-synthetic Test.

1.4 Permutation Test

To test whether the four identified patterns are reproducible and disease-related, we conducted a permutation test for Adjusted Random Index (ARI). Specifically, our null hypothesis is that the patterns will not be reproducible with low ARIs if the samples do not contain distinct clusters with different distribution from 'CN'. To do so, we utilized the whole discovery set (i.e., 297 CN and 602 MCI/Dementia participants from ADNI2/GO) and randomly shuffled the labels of 'CN' and 'PT' to create a null dataset with 297 new 'CN' and 602 'PT' who have comparable distribution in ROI volumes. For each the permuted null dataset, we then trained a new set of mappings from 'CN' to 'PT' and estimated cluster labels by running the hold-out cross validation procedure introduced in the main section, 'Pattern Memberships and Probabilities Assignments', and calculated the mean pair-wise ARI values. A detailed procedure for the Permutation Test can be found in Algorithm 2. For predefined numbers of clusters (M=3-5), we repeated the process 1000 times to derive the null distribution of ARIs for each M. Observed ARIs were calculated by running the inner loop of Algorithm 2 with not shuffled CN and PT data (i.e., data with true 'CN' and 'PT' label assignments). These observed ARIs were then compared with the null ARIs to generate the permutation p-value as the proportion of null ARIs greater than the true ARI over 1000 repetitions. A small p-value indicates that the identified clusters are significantly more reproducible than when normal control and patient are sampled from the same distribution.

Algorithm 2: Permutation Experiment Procedure

for i from 1 to 1000 **do**
    Randomly shuffle diagnosis labels of all 899 participants;
    Select out the new 'CN' group and 'PT' group;
    for *j from 1 to 10* **do**
        Randomly sample 80% of participants from 'CN' and 'PT' group respectively
        Run Smile-GAN model with 80% sampled 'PT' and 'CN' data and derive cluster labels for all 'PT' data;
    end
    Calculate pairwise ARIs among 10 lists of derived labels and saved the mean value as the ARI for the ith permutation;
end

1.5 Derivation of Patterns Among Participants out of ADNI2/GO Study

To further test the reproducibility of four patterns derived from the ADNI2/GO group, we did three different experiments for external validation. (1) We applied 30 models derived from ADNI2/GO dataset to assign pattern probabilities to the baseline data of participants from ADNI1 only. (2) We retrained the model on another independent discovery set consisting of baseline data of 229 CN and 590 MCI/Dementia participants from ADNI1 study and tested on ADNI1 data. (3) We utilized the data from ISTAGING consortium which includes several different studies: PENN-PMC and Open Access Series of Imaging Studies (OASIS-3), which are single-site memory clinic studies,

Australian Imaging, Biomarker & Lifestyle Flagship Study of Ageing (AIBL), Biomarkers of Cognitive Decline Among Normal Individuals (BIOCARD), and BLSA, community studies of aging, the Aging Brain Cohort (ABC), which is a multisite memory center cohort and ADNI1. Baseline T1-MRI data of participants from seven different studies where preprocessed and harmonized following the same procedure introduced in the main section, 'MRI Data Acquisition and Processing'. As the ISTAGING data set was larger, we imposed a stricter selection criterion and used only participants with age greater than 65 and CN participants with Mini-Mental State Exam (MMSE) score greater than 28 to generate a CN group with good cognitive performance. Detailed distribution of participants can be found in Supplementary Table 3. The Smile-GAN model was trained on selected data and clustered them into four subgroups. AFNI 3dttest with GM tissue maps was then performed between CN and each subgroup for all three experiments introduced above to evaluate for similarities and differences across training groups.

Supplementary Table 3: Distribution of participants for external validation

	ADNI1	PENN-PMC	OASIS-3	AIBL	ABC	BLSA	BIOCARD
CN	179	36	414	375	96	443	158
MCI/Dementia	543	437	200	187	51	21	17

1.6 Derivation of Patterns Among Abeta+ MCI/Dementia Participants

To derive patterns observed specifically in Abeta+ participants, we selected out 145 CN Abeta- participants and 317 MCI/Dementia Abeta+ participants from the original ADNI2/GO discovery set to construct the new training set. We reran the Smile-GANs model 30 times and assigned pattern probabilities to all Abeta+ participants using the same procedure introduced in the main section, 'Pattern Memberships and Probabilities Assignments'.

1.7 Comparison with Established Subtypes

We further explored the relationship between our four patterns and subtypes derived in previous MRI-based clustering studies[6]. Limbic predominant (LP), hippocampal sparing (HpSp) and AD-typical (tAD) subtypes have been defined in several MRI-based clustering studies[6-8] as well as pathology-based studies[9]. We utilized baseline data of all 2832 ADNI/BLSA participants and regrouped them into these three subtypes using the straightforward method based on ROI ratio presented in Risacher et al[10]. Risacher et al. defines the limbic predominant group as participants with cortical volume (CTV) /hippocampal volume (HV) > 75th quartile *and* HV < Median HV *and* CTV > Median CTV. The hippocampal sparing group consists of participants with CTV/ HV < 25th quartile *and* HV > Median HV and CTV < Median HV while the tAD group contains the remainder. Besides using the exact same criteria, in a second experiment we also relaxed the criteria by removing the constraint with median volumes and by setting thresholds at the 35th and 65th percentiles.

In addition, a subcortical subtype was identified in several MRI-based unsupervised clustering studies[7,8], but not separately clustered using Smile-GAN. We hypothesized that atrophy variations contributing to this subtype might not be strongly disease related. Therefore, we specifically tested the presence of atrophy in ROIs contributing to this subtype reported in previous studies including Thalamus, Pallidum, Putamen, Caudate and Cerebellum. We selected baseline data of all 899

ADNI2/GO participants used for discovering patterns. ROIs were then residualized by age, sex and ICV effects using a linear regression model to remove effects of these common covariates. Next, we calculated z-scores of ROIs' volumes among the PT group with respect to mean and standard deviation of the CN group. We ranked all ROIs based on the mean Z scores and selected out regions contributing the subcortical subtype as well as the top 18 ROIs for reference. Also, one way ANOVA analysis was performed to compare the CN and PT group in each corrected ROI.

1.8 Prediction of Longitudinal MRI Progression Pathways

P2 and P3 pattern probabilities were directly used for predicting longitudinal pattern stability or pattern progression pathways for P1 participants without special design of the model. Detailed experiments are introduced in the main section, 'Evaluation of Patterns' Predictive Ability'. Participants with both P2 and P3 lower than optimized thresholds were predicted to remain in P1. For participants who have both P2 and P3 over thresholds, the progression direction was predicted by the pattern with higher probability. Only participants with P1 probability greater than 0.7 are included for analysis to avoid trivial prediction cases that would otherwise augment the apparent predictive ability.

1.9 Comparison of P4 participants from Both Progression Pathways

To compare P4 participants arriving along two different pathways, we utilized longitudinal data and focused on participants who had at any timepoint reached P2 or P3 and subsequently P4 (probability of >0.5 was used as threshold for group membership). We selected and regrouped their later visits based on P4 probabilities, obtaining three subgroups with 0.5<P4<0.7, 0.7<P4<0.9, and P4>0.9 for each progression path respectively. AFNI 3dttest with GM tissue maps was then performed between CN and each subgroup.

2. SUPPLEMENTARY RESULTS

2.1 Smile-GAN Model Validation

2.1.1 Synthetic Test

By assigning participants to the pattern type with the highest probability, the Smile-GAN model was able to cluster participants with 100% accuracy even with very severe confounding patterns. Moreover, Supplementary Fig. 1a shows that mapping function f is able to perfectly capture three simulated atrophy patterns while avoiding all non-disease-related patterns.

Supplementary Fig. 1b shows the change of WD and clustering error (i.e., 1 - clustering accuracy) during the training procedure. Generally, the two metrics are consistent in monitoring the training process. Therefore, WD could be used as a surrogate of clustering accuracy when the latter is not available in real applications. Note that inconsistencies at the beginning of training or oscillations exist. Such circumstances can be filtered out by the other two metrics, alteration quantity (AQ)

and cluster loss. We propose to use WD, along with AQ and cluster loss, as metrics for monitoring the training process.

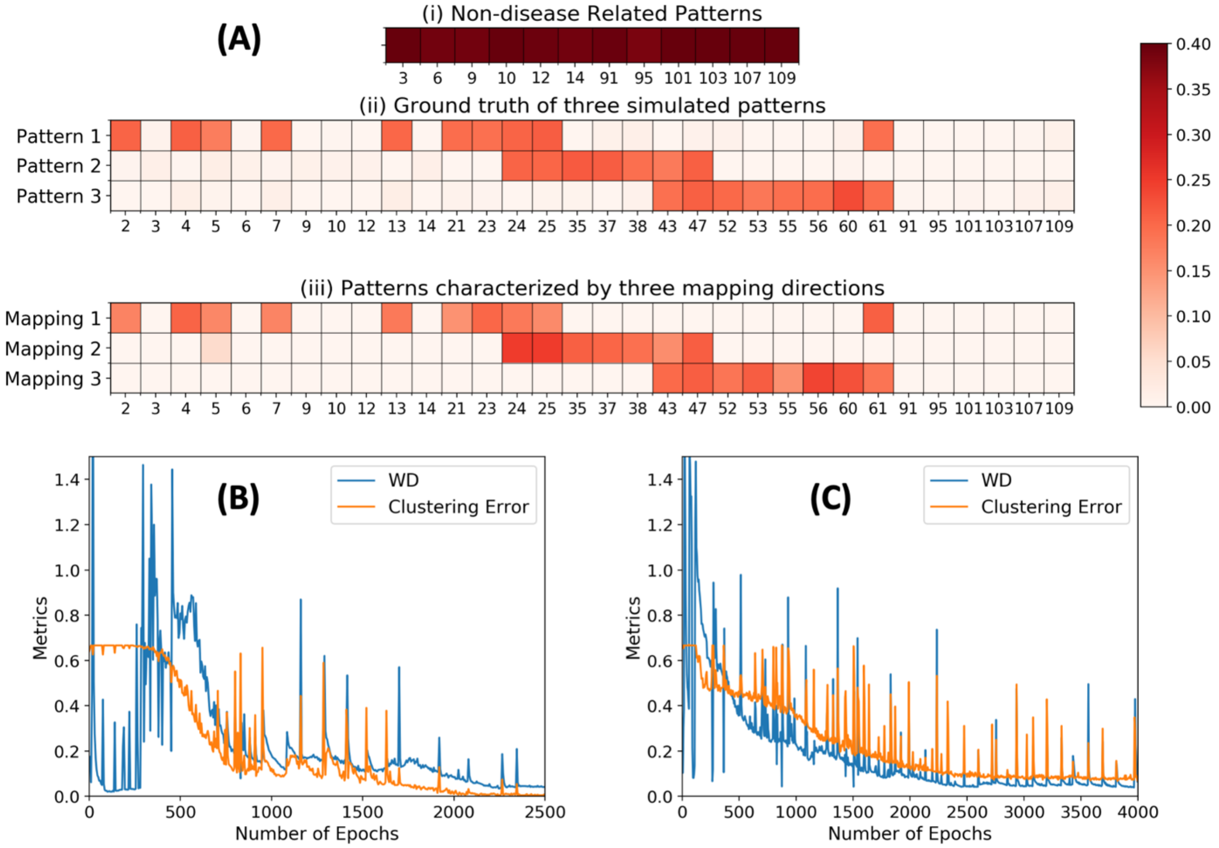

Supplementary Figure 1: Performance of Smile-GAN model on synthetic and semi-synthetic dataset. (a): Performance of model on synthetic data set (supplementary section 1.3.1): The mapping function (f) of Smile-GAN discovers (iii) the ground truth of three simulated atrophy patterns (ii) while not confounded by very severe non-disease related patterns (i). Each mapping direction captures one type of pattern. Only the ROIs whose values decreasing over 5% are displayed. Colors indicate ground truth atrophy rate (i and ii) and atrophy rate inferred by the mapping function (iii). (b): Clustering training process monitoring with Wasserstein distance (WD) on synthetic test. Clustering error = 1- clustering accuracy. (c): Clustering training process monitoring with Wasserstein distance (WD) on semi-synthetic test. Clustering error = 1- clustering accuracy.

2.1.2 Semi-synthetic Test

First, from Supplementary Fig. 1c, we can find out that WD is a good metric for training monitoring on data whose distribution is closer to real data. Also, As shown in Supplementary Table 4, Smile-GAN outperforms all other semi-supervised or unsupervised clustering methods in terms of accuracy of prediction in semi-synthetic test. It is more robust to mild atrophy patterns, overlapping atrophy patterns, and non-disease-related covariates compared to these other methods.

Atrophy rate	Smile-GAN	HYDRA	CHIMERA	K-means	GMM	Variant K-means	Variant GMM

**0.3**	**0.999 ±0.001**	0.972 ±0.005	0.582 ±0.013	0.677 ±0.008	0.678 ±0.000	0.988 ±0.000	0.988 ±0.000
**0.1-0.3**	**0.958 ±0.009**	0.877 ±0.037	0.381 ±0.006	0.369 ±0.004	0.503 ±0.014	0.665 ±0.006	0.738 ±0.015
**0.1-0.2**	**0.825 ±0.017**	0.550 ±0.045	0.371 ±0.004	0.363 ±0.003	0.364 ±0.003	0.430 ±0.008	0.432 ±0.011

Supplementary Table 4: Clustering accuracy comparison between Smile-GAN and other methods. Best derived clustering accuracies are bolded for each experiment.

2.1.3 Rare Pattern and Non-uniform Z Experiment

As shown in Supplementary Table 5, for each experiment with different synthesized atrophy rates (each row in Supplementary Table 5), inclusion of one 20% rare pattern/15% rare pattern gradually undermine the clustering accuracy but not significantly affect the clustering performance. However, including one 10% rare patterns or selecting a non-uniform distribution of Z variable does significantly affect the performance of the model. These experiments results suggest that our selection of a discrete uniform distribution for the Z variable is better for the current implementation of Smile-GAN, though it may possibly be further improved to better handle rare patterns.

Atrophy rate	Smile-GAN (33% rare pattern)	Smile-GAN (20% rare pattern)	Smile-GAN (15% rare pattern)	Smile-GAN (10% rare pattern)	Smile-GAN (non-uniform z distribution)
0.3	**0.999±0.001**	**0.999±0.001**	**0.999±0.001**	0.988±0.006	0.863±0.007
0.1-0.3	**0.958±0.009**	0.928±0.015	0.908±0.022	0.839±0.035	0.756±0.021
0.1-0.2	**0.825±0.017**	0.776±0.021	0.741±0.032	0.641±0.058	0.592±0.049

Supplementary Table 5: Clustering accuracy of Smile-GAN on rare pattern and non-uniform Z experiments. Best derived clustering accuracies are bolded for each experiment.

2.2 Patterns Identified from Participants outside of the ADNI2/GO study

As shown in Supplementary Fig. 2, four patterns derived from three different external replication experiments are closely correlated with four patterns we derived originally from the ADNI2/GO dataset, which demonstrates the reproducibility of the four patterns and their common applicability to participants with cognitive impairment.

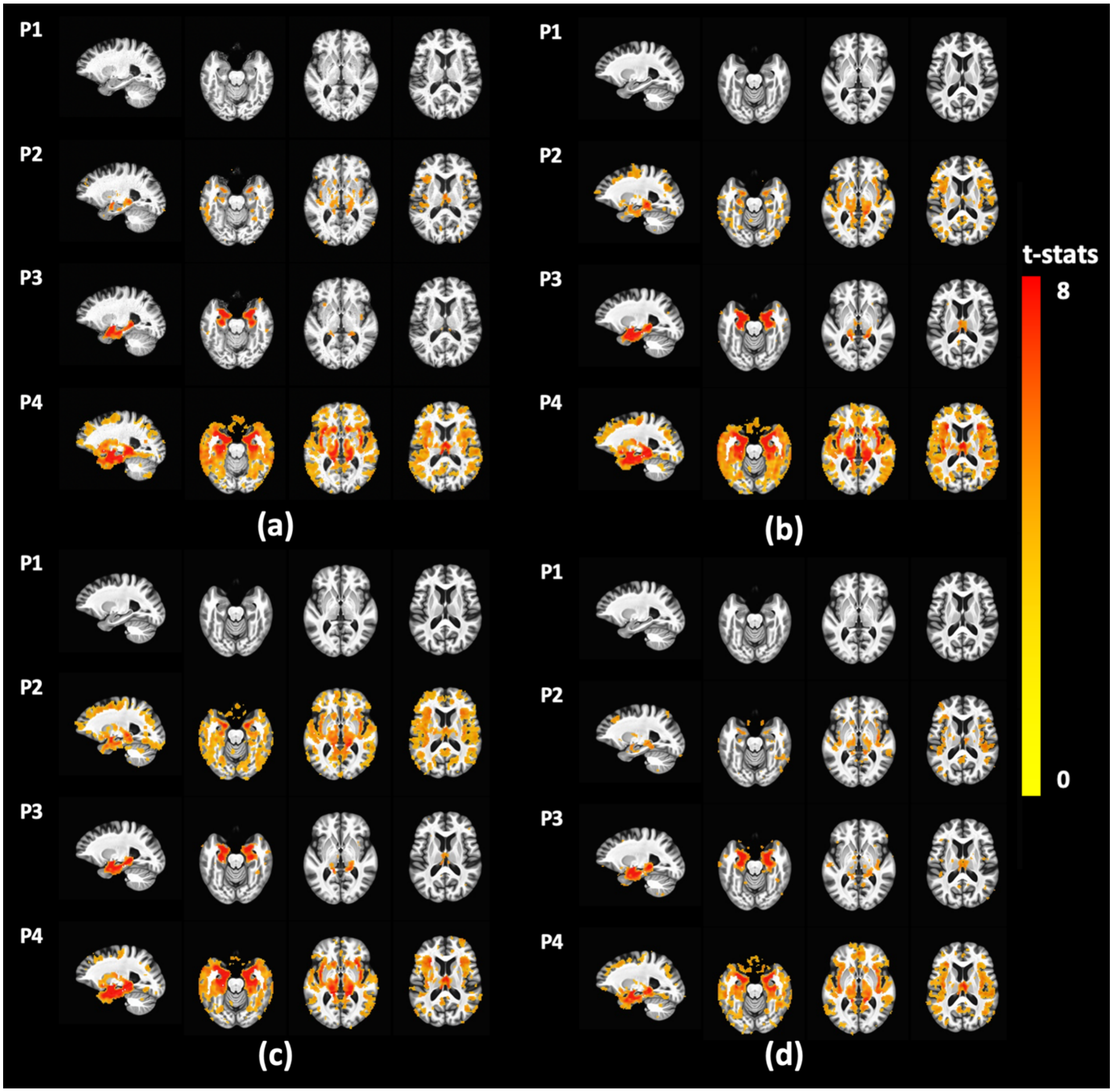

Supplementary Figure 2: Voxel-wise statistical comparison (one-sided t-test) between CN and participants predominantly belonging to four patterns derived from four separate datasets. FDR correction for multiple comparisons with p-value threshold of 0.05 was applied. **(a)** Original four patterns derived from ADNI2/GO dataset and tested on ADNI2/GO dataset; Same as main manuscript Figure 2b **(b)** Experiment 1: four patterns derived from ADNI2/GO dataset and tested on ADNI1 dataset **(c)** Experiment 2: four patterns derived from ADNI1 dataset and tested on ADNI1 dataset. **(d)** Experiment 3: four patterns derived from and tested on a separate multi-study ISTAGING dataset.

2.3 Patterns Identified from Abeta+ MCI/Dementia Participants

As shown in Supplementary Fig. 3a, four patterns derived using only Abeta+ participants (referred as Abeta+ patterns) closely match the original four patterns, except with mildly worsened severity of atrophy across P2-P4. Thus, the original 4 pattern system was able to express patterns observed

in Abeta+ participants, since the four Abeta+ patterns are only shifted forward to a more severe stage from the original four patterns (Supplementary Fig. 3b).

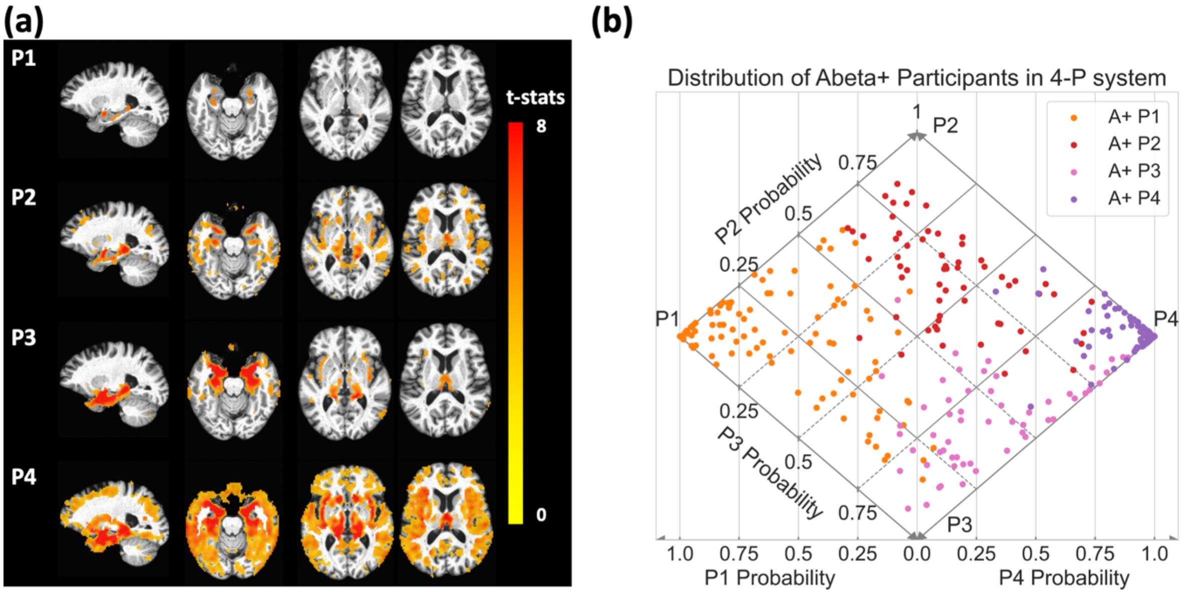

Supplementary Figure 3: Four Abeta+ patterns and their connection with original four patterns. (a) Voxel-wise statistical comparison (one-sided t-test) between CN and Abeta+ participants predominantly belonging to the four Abeta+ patterns. FDR correction for multiple comparisons with p-value threshold of 0.05 was applied. (b) Visualization of Abeta+ participants' expression of original four patterns in the same diamond plot. Colors of each dot are determined by the predominant Abeta+ pattern.

2.4 Permutation Test

Results of the permutation test are shown in Supplementary Fig. 4, which displays the distribution of ARIs for the permuted dataset (blue) and the true observed ARI for M=3-5 (red). For M=3-5, there were significant reproducibility (permutation p values≤0.001 for all). However, for M=4, ARI=0.48 outperforms ARIs for M=3 or 5. Therefore, we chose M=4 as the number of clusters for model training and follow-up analysis. Also, from the permutation test, we can infer that even though there were substantial variations among the PT group, the model did not stably capture them as long as the same variations were also present in the CN group. These results suggest that the four patterns derived by the Smile-GAN model are disease-related and significantly reproducible.

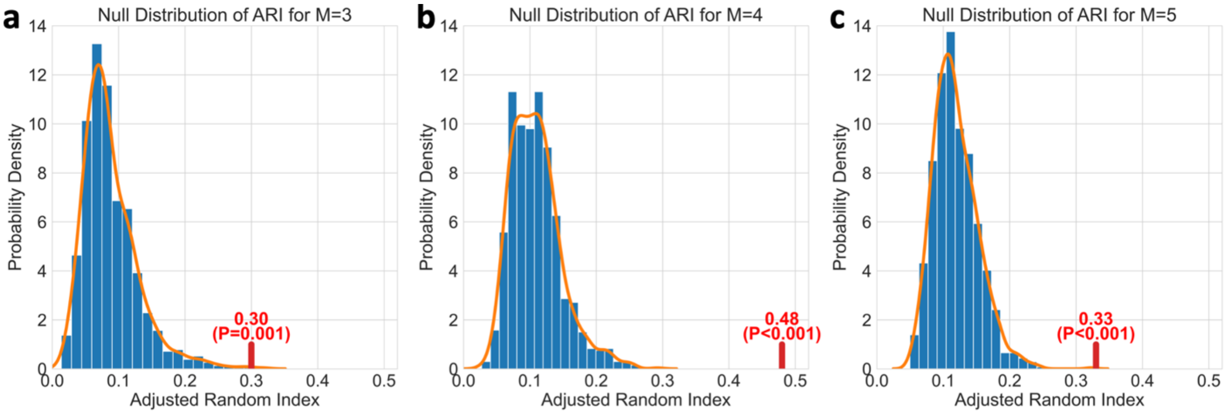

Supplementary Figure 4: Null distributions of ARIs under permutation test with observed ARI for M=3-5. Normalized histograms of null ARIs are plotted (blue) with kernel density estimation (orange). Observed ARI is displayed with the red bar. The permutation p-value equals the proportion of null ARI greater than the observed ARI over 1000 repetitions.

2.5 Comparison with Established Subtypes

As visualized in Supplementary Fig. 5, we observe that limbic predominant (LP) participants mainly fall along the 1-3-4 path and hippocampal sparing (HpSp) participants mainly fall along the 1-2-4 path. With relaxed thresholds, participants with high P2 or P3 values become more predominantly HpSp and LP participant, while HpSp participants and LP participants are more skewed towards P1 and P4 respectively.

In order to investigate the presence of subcortical atrophy, we evaluated and compared regional atrophy across ROIs. As shown in Supplementary Fig. 6, caudate and cerebellum, which contribute to subcortical patterns defined in prior studies[7,8], show very slightly negative or even positive Z-scores, indicating no significant disease-related atrophy in these regions. While Putamen and Palladium, other subcortical ROI, do show slightly more negative Z-scores, there are only significant differences between CN and PT in Left Putamen ($p<0.001$) and Right Pallidum ($p=0.011$) and no significant difference in Right Putamen ($p=0.191$) and Left Pallidum ($p=0.276$). Therefore, regarding regions contributing to the subcortical subtype, these observations suggest that some may not show disease related atrophy and/or some volumetric variation might be too small after normalization with respect to the CN group to be detected/clustered by the semi-supervised clustering methods, like Smile-GAN method.

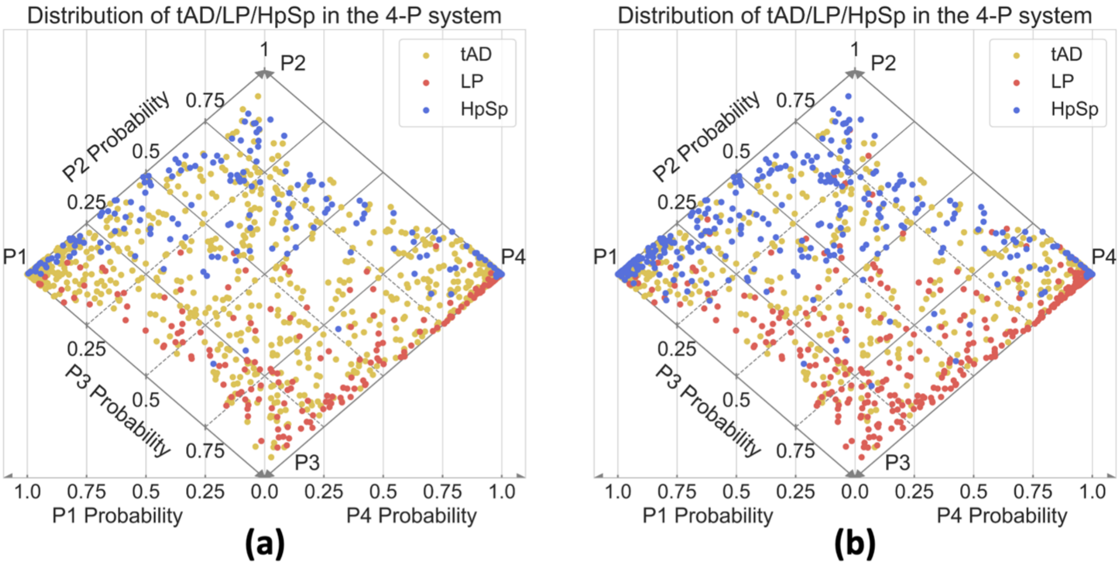

Supplementary Figure 5. Visualization of predicted LP, HpSp and tAD participants in our 4-pattern system. (Data from 2832 ADNI/BLSA participants) Participants are placed on the P1-2-3-4 diamond space as in Figure 2, but are coded based on categorization into tAD, LP, and HpSp phenotypes. These phenotypes are determined by different thresholds: (a) LP: CTV/HV>75 quartile, HV<Median, CTV>Median; HpSp: CTV/HV<25 quartile, HV>Median, CTV<Median (b) LP: CTV/HV>65 quartile; HpSp: CTV/HV<35 quartile.

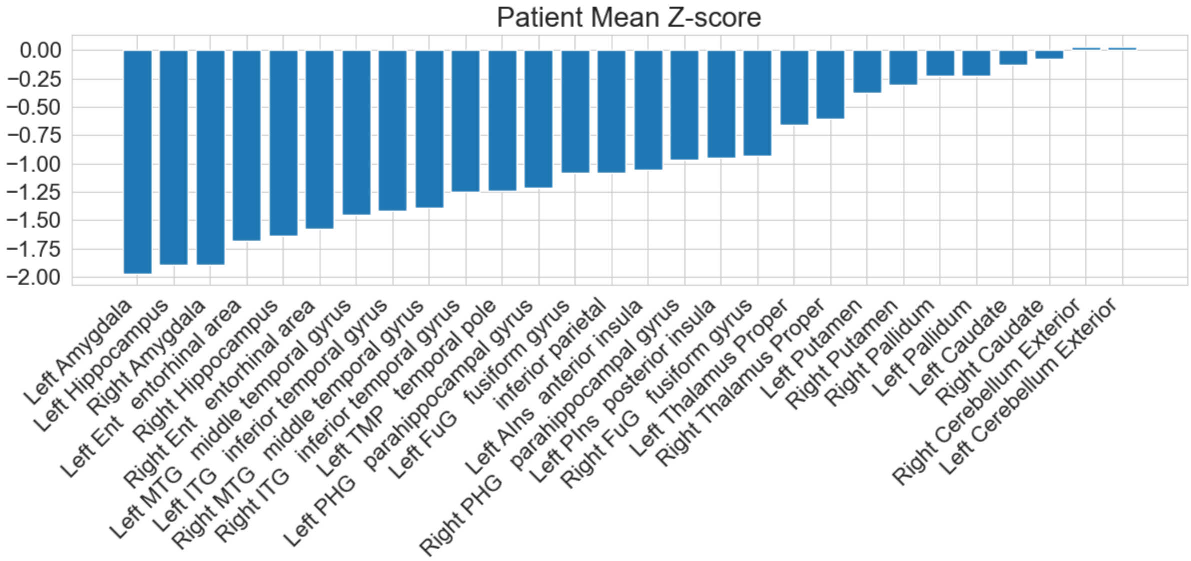

Supplementary Figure 6. (Data from 899 ADNI2/GO participants in discovery set) Mean Z scores of selected ROIs of the PT group with respect to mean and std of CN group.

2.6 Demographics, Clinical biomarkers and Cognitive scores

Clinical characteristics of different groups of participants are revealed in Supplementary Tables 6, 7, and 8.

Table 6 (1)	P1	P2	P3	P4
**TTau**	294.2 (212.0-398.52) (104)	297.5 (204.9-406.9) (117)	341.05 (267.85-446.7) (144)	306.35 (242.58-404.02) (232)
**PTau**	27.52 (20.03-40.19) (104)	30.48 (20.08-40.9) (117)	34.87 (26.11-46.43) (144)	30.3 (23.94-39.36) (232)
**Age**	72.08 (66.74-77.82) (104)	73.68 (66.48-77.66) (117)	73.86 (70.07-77.83) (144)	75.86 (71.15-79.62) (232)
**WML**	34.1 (25.93-49.55) (67)	46.32 (30.18-59.15) (78)	45.13 (33.63-57.24) (75)	50.62 (39.12-66.06) (127)
**ADNI-EF**	0.23 (-0.29-0.97) (86)	-0.29 (-0.82-0.32) (94)	-0.1 (-0.77-0.63) (121)	-0.71 (-1.52--0.11) (190)
**ADNI-MEM**	0.21 (-0.22-0.63) (86)	-0.13 (-0.5-0.41) (94)	-0.39 (-0.75-0.07) (121)	-0.8 (-1.17--0.3) (190)
**ADNI-LAN**	0.26 (-0.15-0.87) (86)	-0.04 (-0.47-0.4) (94)	-0.11 (-0.7-0.44) (121)	-0.62 (-1.34--0.08) (190)
**Hippocampal volume**	0.61 (0.58-0.64) (104)	0.60 (0.57-0.63) (117)	0.54 (0.51-0.56) (144)	0.52 (0.48-0.56) (232)
**Gender (% of male)**	0.61	0.62	0.5	0.63
**% of Tau+**	60.61%	64.35%	78.42%	73.42%
**ApoE ε4 Carriers**	67.31%	52.14%	78.47%	69.83%
**MCI**	92	89	97	102
**Dementia**	12	28	47	130

Table 6 (2)	P1vsP4	P1vsP2	P1vsP3	P2vsP3	P2vsP4	P3vsP4
**TTau**	0.54179	0.99367	**0.0021**	**0.00217**	0.53382	**0.00083**
**PTau**	0.68372	0.53358	**0.00103**	**0.00984**	0.67237	**9.00E-05**
**Age**	**<0.00001**	0.54674	**0.01001**	0.07724	**0.00014**	**0.02783**
**WML**	**2.00E-05**	**0.03131**	**0.0388**	0.86251	**0.04688**	**0.02826**
**ADNI-EF**	**<0.00001**	**<0.00001**	**0.00854**	**0.03826**	**5.00E-05**	**<0.00001**
**ADNI-MEM**	**<0.00001**	**0.00273**	**<0.00001**	**0.00313**	**<0.00001**	**<0.00001**
**ADNI-LAN**	**<0.00001**	**0.00155**	**<0.00001**	0.16643	**<0.00001**	**<0.00001**
**Hippocampal volume**	**<0.00001**	0.16245	**<0.00001**	**<0.00001**	**<0.00001**	**0.02536**
**Gender (% of male)**	0.83049	0.88984	0.12822	0.0603	0.92223	**0.02274**
**% of Tau+**	**0.02988**	0.67273	**0.00451**	**0.01894**	0.10884	0.34426
**ApoE ε4 Carriers**	0.73817	**0.03124**	0.06778	**1.00E-05**	**0.00175**	0.08567

Supplementary Table 6 (1) and (2): Demographics, clinical biomarkers and cognitive scores of participants who are diagnosed as MCI or Dementia and have positive Abeta status at baseline. Hippocampal volume is normalized with respect to 0.01*total brain volume. Median (first quartile – third quartile) are reported. For categorial variables, chi-squared test was used to identify differences between subgroups. For other quantitative variables, a one-way ANOVA analysis was performed for comparison. p-values<0.05 are bolded.

	CN (1)	MCI/Dementia (2)	1 vs 2
**TTau**	338.3 (255.55-389.55) (19)	346.45 (280.9-446.7) (160)	0.32877
**PTau**	33.43 (25.78-41.19) (19)	35.46 (26.97-46.8) (160)	0.48087
**Age**	76.78 (73.73-80.23) (19)	73.86 (69.71-78.07) (160)	**0.02073**
**Education year**	18.0 (16.5-19.0) (19)	16.0 (13.75-18.0) (160)	**0.00127**
**Hippocampal volume**	0.0055 (0.53-0.57) (19)	0.0054 (0.51-0.56) (160)	**0.0179**
**P3 Probability**	0.53 (0.48-0.61) (19)	0.61 (0.49-0.71) (160)	**0.04762**

Supplementary Table 7: Demographics, clinical biomarkers of participants who express a dominant P3 pattern and have positive Abeta status at baseline. Hippocampal volume is normalized with respect to 0.01*total brain volume. Median (first quartile – third quartile) are reported and all comparisons were performed using a one-way ANOVA test. p-values<0.05 are bolded.

	A-/T- (1)	A+/T- (2)	A+/T+ (3)	1 vs 2	1 vs 3	2 vs 3
**TTau**	199.3 (165.95-229.2) (87)	205.8 (164.65-217.5) (35)	378.3 (310.18-483.15) (64)	0.62469	**<0.00001**	**<0.00001**
**PTau**	17.2 (14.67-19.64) (87)	19.07 (15.68-20.6) (35)	39.08 (31.06-48.14) (64)	0.11036	**<0.00001**	**<0.00001**
**Age**	67.95 (62.83-73.66) (87)	71.87 (65.33-78.07) (35)	72.6 (67.48-77.82) (64)	**0.04457**	**0.00829**	0.90376
**WML**	32.0 (22.37-42.05) (67)	31.3 (25.1-51.92) (27)	38.2 (26.65-49.06) (34)	0.09985	**0.02316**	0.75292
**ADNI-EF**	0.71 (0.19-1.37) (71)	0.65 (-0.01-1.08) (28)	0.07 (-0.39-0.54) (53)	0.19943	**2.00E-05**	**0.02983**
**ADNI-MEM**	0.56 (0.18-1.22) (71)	0.38 (0.03-0.74) (28)	0.02 (-0.34-0.52) (53)	0.09148	**<0.00001**	**0.02775**
**ADNI-LAN**	0.56 (0.07-1.27) (71)	0.46 (0.06-0.87) (28)	0.18 (-0.21-0.6) (53)	0.35996	**0.00563**	0.12921
**Hippocampal volume**	0.63 (0.59-0.66) (87)	0.61 (0.59-0.65) (35)	0.6 (0.56-0.63) (64)	0.71596	**0.00032**	**0.01142**
**P1 Probability**	0.84 (0.66-0.95) (87)	0.87 (0.8-0.97) (35)	0.74 (0.57-0.91) (64)	**0.01199**	**0.02842**	**6.00E-05**
**Gender (% of male)**	48.28%	71.43%	57.81%	0.03371	0.31993	0.26209
**ApoE ε4 Carriers**	18.39%	51.43%	75.00%	**0.00054**	**<0.00001**	**0.03112**
**MCI**	84	34	54			
**Dementia**	3	1	10			

Supplementary Table 8: Demographics, clinical biomarkers and cognitive scores of participants who express a dominant P3 pattern and diagnosed as MCI or Dementia at baseline. Hippocampal volume is normalized with respect to 0.01*total brain volume. Median (first quartile – third quartile) are reported. For categorial variables, chi-squared test was used to identify differences between subgroups. For other quantitative variables, a one-way ANOVA analysis was performed for comparison. (A: Abeta; T: pTau) p-values<0.05 are bolded.

2.7 Prediction Results of MRI Progression Pathways

As also mentioned in the main text, prediction performance of longitudinal pattern progression worsens after the fifth year and optimal thresholds to predict progression along either pathway decrease with time. Moreover, among participants correctly classified as progression in either direction, there is a fraction of them who have both P2 and P3 over thresholds as shown in Supplementary Table 9 and, thus, their true progression pathway cannot be clearly revealed by optimal thresholds. For these participants, the true progression direction can be simply indicated by the pattern with higher probability with high accuracies.

	2y	3y	4y	5y	6y	7y	8y
**P1-P2**							

**AUC**	0.857	0.882	0.851	0.848	0.843	0.824	0.807
**Threshold**	0.141	0.128	0.106	0.081	0.078	0.078	0.065
**Accuracy**	0.868	0.849	0.791	0.751	0.753	0.745	0.701
**P1-P3**							
**AUC**	0.904	0.974	0.900	0.907	0.855	0.845	0.839
**Threshold**	0.078	0.080	0.058	0.044	0.037	0.037	0.034
**Accuracy**	0.903	0.916	0.852	0.830	0.779	0.766	0.749
**Participants with both P2 and P3 over thresholds**							
**Quantity**	14.3%	9.7%	21.4%	27.1%	27.6%	29.3%	34.5%
**Accuracy**	100%	100%	100%	93.8%	95.2%	95.8%	90%

Supplementary Table 9: Prediction results of longitudinal progression pathways. AUC and optimal thresholds are reported as described in supplementary section 1.8; Accuracies for P1-P2 and P1-P3 predictions at different time points were derived with optimal thresholds. Quantity shows the fraction of participants who are correctly predicted as progression in either direction but actually have both P2 and P3 over corresponding thresholds. Basing prediction on the higher probability between P2 and P3 when both are above threshold results in accuracy as shown in the last row.

2.8 Comparison of P4 Participants Progressing Along the Two Pathways

As P4 probabilities approach 1, both pathways converge to one common pattern type with atrophy that affects similar ROIs, though there are differences in the severity of atrophy in some regions, like the medial temporal lobe (Supplementary Fig. 7).

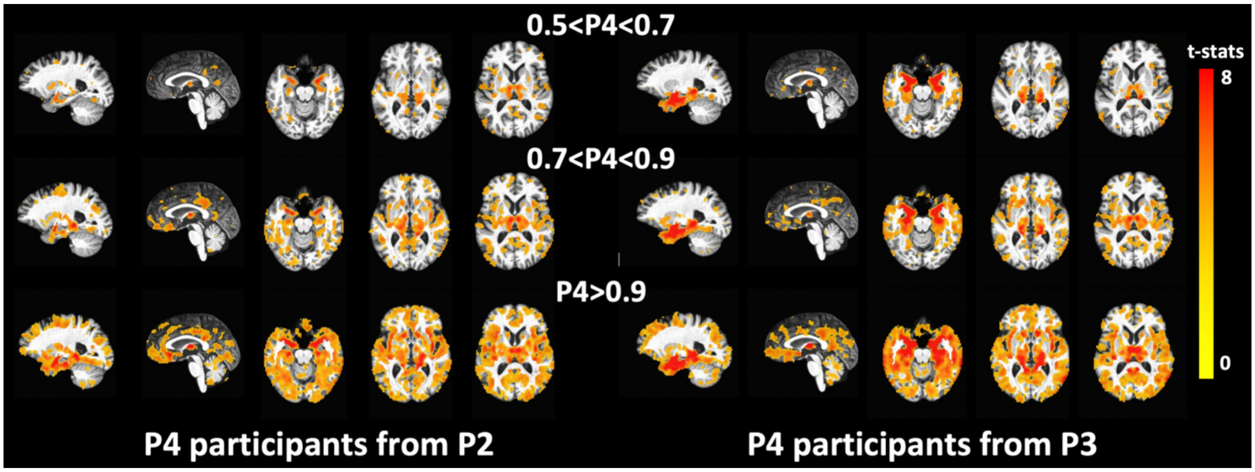

Supplementary Fig. 7: Atrophy development of P4 participants from both pathways. Voxel-wise statistical comparison (one-sided t-test) between CN and 6 subgroups obtained through procedures mentioned in supplementary method section 1.9. FDR correction for multiple comparisons with p-value threshold of 0.05 was applied.

Reference:

1 Chen, X. *et al.* Infogan: Interpretable representation learning by information maximizing generative adversarial nets. *Advances in neural information processing systems* **29**, 2172-2180 (2016).

2 Arjovsky, M., Chintala, S. & Bottou, L. Wasserstein GAN. arXiv:1701.07875 (2017). <https://ui.adsabs.harvard.edu/abs/2017arXiv170107875A>.

3 Kingma, D. P. & Ba, J. Adam: A Method for Stochastic Optimization. arXiv:1412.6980 (2014). <https://ui.adsabs.harvard.edu/abs/2014arXiv1412.6980K>.

4 Varol, E., Sotiras, A., Davatzikos, C. & Alzheimer's Disease Neuroimaging, I. HYDRA: Revealing heterogeneity of imaging and genetic patterns through a multiple max-margin discriminative analysis framework. *NeuroImage* **145**, 346-364, doi:10.1016/j.neuroimage.2016.02.041 (2017).

5 Dong, A., Honnorat, N., Gaonkar, B. & Davatzikos, C. CHIMERA: Clustering of heterogeneous disease effects via distribution matching of imaging patterns. *IEEE transactions on medical imaging* **35**, 612-621, doi:10.1109/tmi.2015.2487423 (2016).

6 Dong, A. *et al.* Heterogeneity of neuroanatomical patterns in prodromal Alzheimer's disease: links to cognition, progression and biomarkers. *Brain : a journal of neurology* **140**, 735-747, doi:10.1093/brain/aww319 (2017).

7 Young, A. L. *et al.* Uncovering the heterogeneity and temporal complexity of neurodegenerative diseases with Subtype and Stage Inference. *Nature communications* **9**, 4273, doi:10.1038/s41467-018-05892-0 (2018).

8 Zhang, X. *et al.* Bayesian model reveals latent atrophy factors with dissociable cognitive trajectories in Alzheimer's disease. *Proceedings of the National Academy of Sciences of the United States of America* **113**, E6535-e6544, doi:10.1073/pnas.1611073113 (2016).

9 Murray, M. E. *et al.* Neuropathologically defined subtypes of Alzheimer's disease with distinct clinical characteristics: a retrospective study. *Lancet Neurol* **10**, 785-796, doi:10.1016/S1474-4422(11)70156-9 (2011).

10 Risacher, S. L. *et al.* Alzheimer disease brain atrophy subtypes are associated with cognition and rate of decline. *Neurology* **89**, 2176-2186, doi:10.1212/wnl.0000000000004670 (2017).
