# Supplementary material for: A deep learning framework identifies dimensional representations of Alzheimer’s Disease from brain structure
Source: Nat Commun. 2021 Dec 3;12:7065. doi: 10.1038/s41467-021-26703-z (PMC8642554; doi:10.1038/s41467-021-26703-z)
Supplement: Supplementary file 3 — Reporting Summary [file 41467_2021_26703_MOESM3_ESM.pdf]

nature research

Corresponding author(s): Christos Davatzikos

Last updated by author(s): Oct 1, 2021

Reporting Summary

Nature Research wishes to improve the reproducibility of the work that we publish. This form provides structure for consistency and transparency in reporting. For further information on Nature Research policies, see our Editorial Policies and the Editorial Policy Checklist.

Statistics

For all statistical analyses, confirm that the following items are present in the figure legend, table legend, main text, or Methods section.

n/a	Confirmed	
☐	☒	The exact sample size (*n*) for each experimental group/condition, given as a discrete number and unit of measurement
☐	☒	A statement on whether measurements were taken from distinct samples or whether the same sample was measured repeatedly
☐	☒	The statistical test(s) used AND whether they are one- or two-sided<br>*Only common tests should be described solely by name; describe more complex techniques in the Methods section.*
☐	☒	A description of all covariates tested
☐	☒	A description of any assumptions or corrections, such as tests of normality and adjustment for multiple comparisons
☐	☒	A full description of the statistical parameters including central tendency (e.g. means) or other basic estimates (e.g. regression coefficient) AND variation (e.g. standard deviation) or associated estimates of uncertainty (e.g. confidence intervals)
☐	☒	For null hypothesis testing, the test statistic (e.g. *F*, *t*, *r*) with confidence intervals, effect sizes, degrees of freedom and *P* value noted<br>*Give P values as exact values whenever suitable.*
☒	☐	For Bayesian analysis, information on the choice of priors and Markov chain Monte Carlo settings
☒	☐	For hierarchical and complex designs, identification of the appropriate level for tests and full reporting of outcomes
☒	☐	Estimates of effect sizes (e.g. Cohen's *d*, Pearson's *r*), indicating how they were calculated

Our web collection on statistics for biologists contains articles on many of the points above.

Software and code

Policy information about availability of computer code

Data collection	N4BiasCorrection: https://github.com/ANTsX/ANTs/releases/tag/v2.3.1; MASS: https://github.com/CBICA/MASS/releases/tag/1.1.1; MUSE: https://github.com/CBICA/MUSE/releases/tag/3.0.5 were used for MRI data preprocessing. Software for ROI-wise pattern type clustering (Smile-GAN model) is available as a published PyPI package SmileGAN 0.1.0. Detailed requirement and instruction on implementation can be found at: https://pypi.org/project/SmileGAN/. Custom code for SmileGAN can be found at: https://github.com/zhijian-yang/SmileGAN.
Data analysis	Code for data analysis were based on online python packages including scikit-learn 0.20.4; numpy 1.16.6; pandas 0.21.0; statsmodels 0.8.0; lifelines 0.25.7. Online python package scikit-learn 0.20.4; pyHYDRA 1.0.8; CHIMERA: https://github.com/aoyandong/CHIMERA were used for model comparisons.

For manuscripts utilizing custom algorithms or software that are central to the research but not yet described in published literature, software must be made available to editors and reviewers. We strongly encourage code deposition in a community repository (e.g. GitHub). See the Nature Research guidelines for submitting code & software for further information.

Data

Policy information about availability of data

All manuscripts must include a data availability statement. This statement should provide the following information, where applicable:

- Accession codes, unique identifiers, or web links for publicly available datasets
- A list of figures that have associated raw data
- A description of any restrictions on data availability

Data used for this study was provided from ADNI and BLSA studies via data sharing agreements that did not include permission to further share the data. Data from ADNI are available from the ADNI database (adni.loni.usc.edu) upon registration and compliance with the data usage agreement. Data from the BLSA are available upon request from the BLSA website (blsa.nih.gov). All requests are reviewed by the BLSA Data Sharing Proposal Review Committee and may also be subject to

approval from the NIH Institutional Review Board. Those interested in accessing study data or derived imaging variables used in this study may seek approval from studies. If granted, we would be able to provide participant-level derived imaging variables used in this study within 1 month of approval. Source data are provided with this paper.

Field-specific reporting

Please select the one below that is the best fit for your research. If you are not sure, read the appropriate sections before making your selection.

☒ Life sciences ☐ Behavioural & social sciences ☐ Ecological, evolutionary & environmental sciences

For a reference copy of the document with all sections, see nature.com/documents/nr-reporting-summary-flat.pdf

Life sciences study design

All studies must disclose on these points even when the disclosure is negative.

Sample size	All participants from ADNI1, ADNI2/GO and BLSA study were included for the analysis. Detailed description of studies, participants and sample sizes can be found in the section 4.2 in the main manuscript.
Data exclusions	Participants without required clinical variables for specific analyses were excluded for analyses. Detailed description of included participants and required clinical variables can be found in different methods sections for different analyses.
Replication	The reproducibility of pattern type clustering is tested through a 10% leave-out cross validation and evaluated by Adjusted Random Index as described in the section 4.6. All other longitudinal prediction tasks are implemented through a 2-fold cross validation as described in section 4.8.
Randomization	For 10% leave-out cross validation, we randomly left out 10% of training data each time running the Smile-GAN model. For longitudinal prediction tasks, we randomly half divided data into training and validation set each time training and testing the model.
Blinding	No data collection was performed for this study. There was no blinding during data analysis. Study methods were either observational with respect to participant classification or used randomized sampling and cross-validation without direct user input.

Reporting for specific materials, systems and methods

We require information from authors about some types of materials, experimental systems and methods used in many studies. Here, indicate whether each material, system or method listed is relevant to your study. If you are not sure if a list item applies to your research, read the appropriate section before selecting a response.

Materials & experimental systems

n/a	Involved in the study	
☒	☐	Antibodies
☒	☐	Eukaryotic cell lines
☒	☐	Palaeontology and archaeology
☒	☐	Animals and other organisms
☐	☒	Human research participants
☐	☒	Clinical data
☒	☐	Dual use research of concern

Methods

n/a	Involved in the study	
☒	☐	ChIP-seq
☒	☐	Flow cytometry
☐	☒	MRI-based neuroimaging

Human research participants

Policy information about studies involving human research participants

Population characteristics	Table 1 in the main manuscript provides the Information of participants from different studies including diagnosis, gender, age, genotype and availability of other clinical characteristics.
Recruitment	This study was a retrospective analysis. There was no novel participant recruitment.
Ethics oversight	The study was approved by the Institutional Review Board of the University of Pennsylvania.

Note that full information on the approval of the study protocol must also be provided in the manuscript.

Clinical data

Policy information about clinical studies

All manuscripts should comply with the ICMJE guidelines for publication of clinical research and a completed CONSORT checklist must be included with all submissions.

Clinical trial registration	Not applicable; not a prospective clinical trial. ADNI (NCT01231971, NCT00106899) and BLSA (NCT00233272) are listed on clinicaltrials.gov.
Study protocol	Not applicable; this study was a retrospective analysis.
Data collection	There was no novel data collection. Data collection methods are defined directly by the ADNI and BLSA studies.
Outcomes	This study does not involve testing a clinical outcome.

Magnetic resonance imaging

Experimental design

Design type	Retrospective cohort study.
Design specifications	This is a retrospective study of data acquired by other research studies. All study data was available at the onset of this research study.
Behavioral performance measures	Clinical measures were obtained from the primary studies (ADNI and BLSA). There was no additional method to validate/verify clinical data.

Acquisition

Imaging type(s)	Structural MRI (T1, FLAIR)
Field strength	1.5 T and 3 T
Sequence & imaging parameters	MRI acquisition was controlled and performed by other studies; no new imaging was performed for this study. Both ADNI and BLSA have previously published methods for data acquisition.
Area of acquisition	A whole brain scan was used

Diffusion MRI ☐ Used ☒ Not used

Preprocessing

Preprocessing software	All image processing tools are publicly available as software packages that can be downloaded from public repositories.<br>N4BiasCorrection: https://github.com/ANTsX/ANTs/releases/tag/v2.3.1<br>MASS: https://github.com/CBICA/MASS/releases/tag/1.1.1<br>MUSE: https://github.com/CBICA/MUSE/releases/tag/3.0.5
Normalization	To generate the tissue density maps (RAVENS maps), we normalized the subject T1 scans to the MNI template and followed the procedure described in [1]. We used a non-linear deformation method [2] to normalize the images.<br>[1] Davatzikos, C., Genc, A., Xu, D. & Resnick, S. M. Voxel-based morphometry using the RAVENS maps: methods and validation using simulated longitudinal atrophy. NeuroImage 14, 1361-1369, doi:10.1006/nimg.2001.0937<br>[2] Ou, Y., Sotiras, A., Paragios, N., & Davatzikos, C. (2011). DRAMMS: Deformable registration via attribute matching and mutual-saliency weighting. Medical image analysis, 15(4), 622-639.
Normalization template	We used the MNI152 template, in the LPS orientation to normalize all subjects to a common space.
Noise and artifact removal	Multi-atlas ROI segmentation method is robust to noise and imaging artifacts because it's based on a consensus labeling approach, and also because it uses a deformable registration algorithm designed to reduce the negative impact of missing correspondences between images. Accordingly we did not need to apply further noise and artifact removal steps.
Volume censoring	Not applicable; Volume censoring is a preprocessing step specific for fMRI data and we don't use a technique equivalent to sMRI data.

Statistical modeling & inference

Model type and settings	Smile-GAN model was applied on ROI volumes to derive pattern probabilities for all participants. Pattern probabilities were used as features for cox proportional hazard model and used to stratify participants for nonparametric survival analysis. Random intercept model with time as fixed effect was applied on ROI volumes to derive annual volume change rate of selected regions.
Effect(s) tested	One way ANOVA analysis was used to compare group differences in single ROI volumes and pattern probabilities.

Specify type of analysis: ☐ Whole brain ☐ ROI-based ☒ Both

Anatomical location(s): 145 anatomical regions of interest (ROIs) were identified using a multi-atlas label fusion method[1] [1]Doshi, J. et al. MUSE: MUlti-atlas region Segmentation utilizing Ensembles of registration algorithms and parameters, and locally optimal atlas selection. Neuroimage 127, 186-195, doi:10.1016/j.neuroimage.2015.11.073 (2016).

Statistic type for inference (See Eklund et al. 2016): Voxel-wise group comparisons (CN vs each pattern) was performed via AFNI 3dttest using voxel-wise tissue density maps (RAVENs maps).

Correction: False discovery rate (FDR) correction for multiple comparisons with p-value threshold of 0.05 was applied.

Models & analysis

n/a	Involved in the study
☒	☐ Functional and/or effective connectivity
☒	☐ Graph analysis
☐	☒ Multivariate modeling or predictive analysis

Multivariate modeling and predictive analysis: Smile-GAN model was applied on ROI data to derive baseline pattern probabilities for participants. Baseline probabilities were then utilized as parts of features sets for longitudinal prediction via cox proportional hazard model. Detailed description of cross-validation procedure and evaluation metric can be found in section 4.8/4.9.
